# Prognosis significance of HER-2/neu overexpression/amplification in Chinese patients with curatively resected gastric cancer after the ToGA clinical trial

**DOI:** 10.1186/1477-7819-10-274

**Published:** 2012-12-18

**Authors:** Fei Zhou, Ning Li, Weihua Jiang, Zhaolai Hua, Lin Xia, Qingyi Wei, Liwei Wang

**Affiliations:** 1 Department of Oncology, Shanghai First People’s Hospital Affiliated Shanghai Jiaotong University, Shanghai, 200080, China; 2 Cancer Institute of Yangzhong City, People’s Hospital of Yangzhong City, Jiangsu Province, 212200, China; 3 Department of Pathology, People’s Hospital of Yangzhong City, Jiangsu Province, 212200, China; 4 Department of Epidemiology, University of Texas MD Anderson Cancer Center, Houston, TX, USA

**Keywords:** FISH, Gastric cancer, HER-2/neu, IHC, Prognosis

## Abstract

**Background:**

HER-2/neu-targeted therapy has been successfully used in advanced gastric cancer, but the role of HER-2/neu in the prognosis of gastric cancer is not yet clear. In this study, we investigated the correlation between HER-2/neu expression and amplification as well as their association with clinic outcomes in patients with curatively resected gastric cancer.

**Methods:**

We constructed tissue microarray blocks containing >70% of gastric cancer tissue and matched adjacent normal gastric tissue for 227 patients. Expression of the HER-2/neu protein in these specimens was analyzed using immunohistochemical staining. Amplification of HER-2/neu was also analyzed for the same samples using fluorescence *in situ* hybridization. Data on clinicopathological features and relevant prognostic factors in these patients were analyzed.

**Results:**

Of the 227 gastric cancer samples, 11.89% were positive for HER-2/neu overexpression/amplification under the new scoring system. HER-2/neu overexpression/amplification was closely correlated to the Lauren type, degree of differentiation, tumor size and lymph node metastasis. HER-2/neu overexpression/amplification predicted poor survival in univariate analysis but not in a Cox proportional hazards model.

**Conclusion:**

HER-2/neu overexpression/amplification was not an independent predictor for survival in patients with curatively resected gastric cancer.

## Background

Although the incidence of gastric cancer is decreasing worldwide, it is still relatively high in China with 420,000 newly diagnosed patients each year. The mortality rate of gastric cancer in China ranks third among all cancer deaths, with 320,000 patients for the period of 2004 to 2005 [1]. Although there has been great improvement in the early diagnosis of gastric cancer, in combination with the recent progress of surgical techniques and comprehensive use of chemotherapy and radiotherapy, the 5-year survival rate for gastric cancer remains as low as 20% to 30% [2].

Targeted therapy is a new trend in cancer treatment to improve overall survival in patients with cancer. In regard to gastric cancer, molecularly targeted therapy is also gaining status. Antiangiogenic therapy and anti-epidermal growth factor receptor therapy have emerged as a new hope [3], especially the anti-HER-2 drug, trastuzumab. According to the ToGA clinical trial reported in 2010 [4], patients with HER-2/neu overexpression receiving chemotherapy and trastuzumab had a significant longer median overall survival of 13.8 months without any additional adverse side effects. Shitara *et al*. also reported that patients who were HER-2-positive with advanced gastric cancer had a better prognosis than patients who were negative for HER-2 when treated with trastuzumab [5]. All these indicate that the HER-2/neu-targeted therapy has great potential in improving the treatment of gastric cancer.

Oncogene HER-2/neu was originally discovered in chemically induced rat neuroglioblastomas [6] and is located on chromosome 17q21 and codes for a transmembrane glycoprotein of 185kD. The protein consists of an extracellular ligand-binding domain of 653 amino acids, a single membrane-spanning region of 654 to 675 amino acids, and a cytoplasmic protein tyrosine kinase domain of 675 to 1,255 amino acids. It is one of the members of the human epidermal growth factor receptor (HER) family that includes HER-1 (epidermal growth factor receptor), HER-2/neu, HER-3 and HER-4 [7]. Among these, HER-2/neu plays an important role in normal development, differentiation and apoptosis of the cell. Gene amplification and overexpression of HER-2/neu have been reported in many cancer types, including that of the ovary [8], lung [9] and prostate [10], in addition to the breast [11]. For example, it has been shown that HER-2/neu overexpression was detected in about 10% to 34% of invasive breast cancers and was associated with poor prognosis. Although HER-2/neu is considered an independent prognostic factor for breast cancer, its prognostic role in gastric cancer still remains controversial because several studies have generated conflicting results [12-16].

To assess the association between the status of Her-2/neu and prognosis of Chinese patients with gastric cancer, we conducted a (we think “the” is better, it means this study) study to examine both expression and amplification of Her-2/neu in tumors of curatively resected gastric cancer and correlated these measurements to the clinicopathological and prognostic outcomes of the patients.

## Methods

### Patients and clinicopathological information collection

A total of 227 patients with gastric cancer from the pathology archives of the Yangzhong People’s Hospital were included in this study between 2002 and 2004. All of the patients (except those classified as T1N0M0) had undergone curative surgery and received a similar adjuvant regimen of chemotherapy (5-fluorouracil and cisplatin). Clinicopathological variables including age, sex, histologic type and pathologic stage were collected by reviewing medical charts and pathology records. Among these patients, 157 were men and 70 were women, with an age range between 24 and 79 years old (a median of 60 years old). Tumor sites included 28 antrum, 50 corpus and 149 cardia. Patients were followed from the date of surgery until death, or censored on 31 December 2008, which resulted in a follow-up period of 1 to 108 months (a median of 64 months). The study was approved by our Institutional Ethnic Committee (equivalent to an institutional review board). As this was a retrospective study using archive tissue specimens, the Institutional Ethnic Committee waived the need for written informed consent.

### Tissue microarray construction

In brief, H & E-stained sections were made from primary tumor blocks to define two representative tumor regions and adjacent normal gastric tissues. Representative tumor regions were defined as tumor solid areas containing more than 75% cancer cells without necrosis. Normal gastric tissues were randomly selected adjacent to a tumor with a distance of more than 5 cm, avoiding the bleeding areas. Tumor typing and grading were performed according to Lauren and the World Health Organization (WHO) criteria. All patients with gastric cancer were staged using the seventh edition of the International Union Against Cancer Tumor-Node-Metastasis (TNM) staging system. Tissue cylinders (1.5 mm in diameter) were then punched from the defined regions of the block using a tissue microarrayer (Gentury, IL, USA) and brought into recipient paraffin blocks. Two sets of three paraffin-embedded tissue microarray (TMA) blocks were made. Sections of the resulting TMA blocks were transferred to glass slides. There were a total of two sets of TMA, containing 227 tumor tissue spots and 135 adjacent normal gastric tissue spots each, available for this study (collaborating with Shanghai Biochip, Shanghai, China).

### Immunohistochemistry

Immunohistochemistry (IHC) staining for HER-2/neu was conducted on TMA sections. Formalin-fixed, paraffin-embedded sections were dewaxed in xylene and rehydrated through graded alcohol. Endogenous peroxidase activity was quenched by 3% hydrogen peroxide, then the section was washed in water and the antigen retrieved and placed in citrate buffer. The sections were washed with phosphate-buffered saline, pH 7.2. The primary monoclonal mouse antibody against human HER-2/neu protein (MAB-0198, Maxin Biotech, Fujian, China) was applied for 1 hour in the incubator at 37°C. Anti-HER-2 antibody (Dako REAL, En Vison, HRP Rabbit/Mouse, Dako, Carpinteria, CA, USA) was then applied. Diaminobenzidine solution was used as a chromogen.

### Fluorescence *in situ* hybridization

Fluorescence *in situ* hybridization (FISH) analysis was applied to the sections. Amplification of the *HER*-*2* gene was determined by FISH using a Vysis dual-color, dual-fusion translocation probe set purchased from Abbott Molecular Inc. (DesPlaines, IL, USA). In brief, sections were deparaffinized, dehydrated, and then incubated in 30% sodium bisulfate for 20 min at 45°C. After being washed in 2× SSC Sodium citrate-Hydrochloric acid Buffer solution }, slides were treated with proteinase K at 37°C for 25 min. Then hybridization was carried out overnight at 42°C in a humid chamber, followed by post-hybridization washes in denaturation solution and 0.1% NP-40 with 2×SCC Sodium citrate-Hydrochloric acid Buffer solution }at room temperature. Finally, slides were washed and counterstained with 0.2 μmol/L 4′-6-diamidino-2-phenylindole and examined under a confocal laser scanning microscope LSM 510 (Carl-Zeiss, Jena, Germany).

### Scoring of immunohistochemistry and fluorescence *in situ* hybridization

HER-2 immunostaining was scored using the following scoring system adopted by Hofmann in the ToGA clinical trial: score 0, no membrane staining or <10% of cells stained; 1+, faint/barely perceptible membranous reactivity in 10% of cells or higher or reactivity in only part of the cell membrane; 2+, weak to moderate complete or basolateral membranous reactivity in 10% of tumor cells or higher; and 3+, strong complete or basolateral membranous reactivity in 10% of tumor cells or higher. Scores of 0 and 1+ were considered negative for HER-2/neu overexpression, and scores of 3+ were considered positive. Scores of 2+ were considered overexpression if FISH confirmed amplification [4,17]. Gene amplification was defined as cancer cell nuclei exhibiting a ratio of HER-2/neu to CEP17 (centromeric probe 17) ≥2, or when an HER-2/neu signal cluster was observed.

All samples on the TMA sections from gastric cancers were reviewed by two pathologists independently to determine scores of IHC. For discordant opinions, the samples were re-examined by the two pathologists to achieve a consensus score.

### Statistical analysis

Categorical data were analyzed using *χ*^*2*^ statistics. The probability of survival by different subgroups was calculated using the Kaplan-Meier method, and statistical significance was analyzed by using the log-rank test. Multivariate analysis was carried out by using the Cox proportional hazards model with adjustment for covariates to identify primary prognostic indicators that were independently associated with survival. All statistics were two-sided, at a significant level of *P* <0.05, by using the SPSS statistical software package for Windows (release 13.0, SPSS, Inc., Chicago, IL, USA).

## Results

### HER-2/neu protein expression status and clinicopathological variables in 227 cases of gastric carcinoma

**Figure 1:**
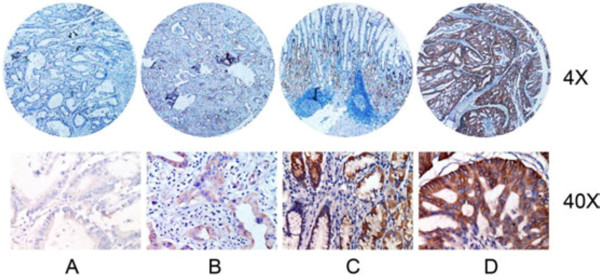
**Immunohistochemical staining for HER-2/neu expression in tumor samples from patients with gastric cancer. (A)** Membrane staining in normal gastric epithelium for HER-2/neu (0+). **(B)** Faint membrane staining in >10% tumor cells (1+). **(C)** Moderate complete membrane staining in >10% tumor cells (2+). **(D)** Strong complete membranes staining in >10% tumor cells (3+). (H&E; original magnification, ×4, ×40).

HER-2/neu protein expression in gastric cancer tissues was determined by IHC for 227 patients. As shown in Figure 1, the immunostaining revealed that expression was detectable only in the cell membranes of tumor cells but not in the adjacent normal gastric epithelial cells. Of these 227 tumors, 189 cases (83.41%) scored 0; 4 cases (1.75%) scored 1+; 11 cases (4.80%) scored 2+; and 23 cases (10.04%) scored 3+. Among 11 cases of 2+, 4 cases (36.4%) were positive for amplification. Therefore, 27 cases (11.89%) had HER-2/neu overexpression. The correlation between HER-2/neu protein expression levels and clinicopathological variables are summarized in Table 1. Compared with tumors without overexpression, tumors of gastric cancer with HER-2/neu overexpression showed predominantly well- or moderately differentiated histology by the WHO classification (*P* <0.05) and an intestinal type histology by the Lauren classification (*P* <0.05). HER-2/neu overexpression was also associated with tumor size and lymph node metastasis (*P* <0.05). However, no differences in the expression levels by age, sex, tumor location, invasion depth, TNM stage, vessel invasion or Borrmann type were found for all (*P* >0.05).

**Table 1:** Comparison of clinicopathological features according to HER-2 overexpression/amplification

**Clinicopathological features**	**HER-2 overexpression/amplification**		
	**HER-2/neupositive (n = 27)**	**HER-2/neunegative (n = 200)**	***P***
Age			0.151
<65 years	18	96	
≥65 years	9	104	
Sex			0.765
male	18	139	
female	9	61	
Lauren			0.001
intestinal	26	118	
diffuse	0	49	
mixed	1	33	
WHO classification			0.001
W/D	9	24	
M/D	16	97	
P/D	2	79	
Tumor size			0.002
≥5 cm	18	72	
<5 cm	9	128	
Tumor location			0.258
cardia	21	128	
corpus	5	45	
antrum	1	27	
Invasion depth			0.322
T1	1	19	
T2	4	32	
T3	22	149	
Lymph node			0.022
present	20	118	
absent	7	82	
TNM stage			0.320
I	3	43	
II	6	52	
III	18	105	
Vessel invasion			0.241
present	4	16	
absent	23	184	
Borrmann			0.133
I	4	26	
II	17	102	
III	6	64	
IV	0	8	

M/D: moderately differentiated; P/D: poorly differentiated; W/D: well differentiated

WHO: World Health Organization.

### HER-2/neu amplification status in 227 cases of gastric carcinoma

**Figure 2:**
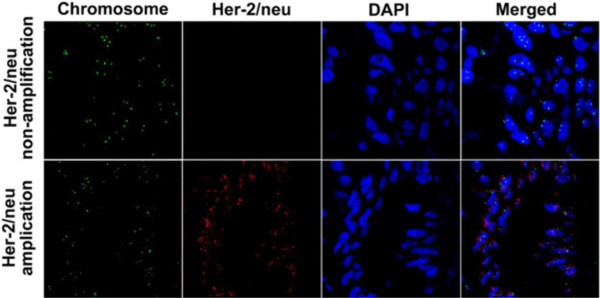
**Fluorescence *in situ *hybridization of HER-2/neu amplification (original magnification, ×60).**

In the FISH analysis, gene amplification was detected in all tumor samples (Figure 2). When the results of FISH and IHC were compared (Table 2), IHC 0+ or 1+ samples did not exhibit amplification (0%), whereas 23 tumors with 3+ immunostaining all showed amplification (100%). Nevertheless, among the 11 tumors with 2+ immunostaining, only showed amplification (36.37%). There were 27 cases (11.89%) of HER-2/neu amplification in the same tumors that had overexpression.

**Table 2:** Correlation between overexpression and amplification for HER-2/neu

**FISH**	**IHC**				
	**0 n = 189**	**1+ n = 4**	**2+ n = 11**	**3+ n = 23**	**Total n = 227**
No amplification	189	4	7	0	227
Amplification	0	0	4	23	27
Amplification rate (%)	0	0	36.40	100	11.89

FISH: fluorescence *in situ* hybridization; IHC: immunohistochemistry.

### Survival analysis

Survival analysis was performed on these 227 patients. Patients with tumors with HER-2/neu overexpression/amplification showed lower 5-year survival rates than those without (27.3% versus 42.8%, *P* <0.05). In our univariate analysis, Lauren classification, differentiated histology, tumor size (≥5 cm), invasion depth, lymph node metastasis, TNM stage, vessel invasion and HER-2/neu overexpression/amplification were all associated with poor survival (*P* <0.05) (Table 3). A final multivariate Cox proportional hazards model identified Lauren classification, vessel invasion, TNM stage and tumor size (≥ 5cm) as bearing prognostic importance (*P* <0.05); however, HER-2/neu overexpression/amplification was not an independent prognostic factor in this model (Table 3).

**Table 3:** Univariate and multivariate overall survival analysis for 227 patients with gastric cancer

**Variables**	**Univariate**		**Multivariate**	
	**5-year survival rate**	***P***	**Hazard ratio**	***P***
Age		0.773		
≥65 years	39.3			
<65 years	41.9			
Sex		0.230		
male	43.3			
female	34.3			
Lauren		<0.001		0.006
intestinal	48.6		1	
diffuse	18.4		1.467	
mixed	38.2		1.201	
WHO classification		0.001		0.003
W/D	63.6			
M/D	45.1			
P/D	24.7			
Tumor size		0.001		
≥5 cm	21.1		1.395	0.005
<5 cm	53.9		1	
Tumor location		0.368		0.647
cardia	40.3			
corpus	35.9			
antrum	50.0			
Invasion depth		<0.001		0.604
T1	85.0			
T2	66.7			
T3	29.8			
Lymph node		<0.001		<0.001
present	25.4			
absent	64.0			
TNM stage		0.007		<0.001
I	76.1		1	
II	51.7		1.364	
III	22.0		1.571	
Vessel invasion		0.001		0.016
present	15.0		1.916	
absent	43.0		1	
Borrmann		0.471		0.221
I	73.3			
II	39.5			
III	32.9			
IV	0			
HER-2 overexpression				0.351
positive	27.3	0.031		
negative	42.8			

M/D: moderately differentiated; P/D: poorly differentiated; W/D: well differentiated.

## Discussion

Since the publication of the ToGA study, the standard for HER-2/neu overexpression has changed from IHC 2+ or 3+ to IHC 3+ or IHC 2+ with amplification, which means that conclusions reached before in the literature should be assessed again and could be different according to the new criteria. In our study, we evaluated HER-2/neu overexpression and amplification in 227 curatively resected gastric cancers using the new criteria. We performed IHC and FISH on all the samples and found that all the cases of 3+ showed amplification, 4 of 11 cases of 2+ showed amplification, but all the cases of 0+ and 1+ exhibited no amplification. Because there was no amplification in IHC 0+ and 1+, the cases of tumors with HER-2/neu amplification equals the cases of tumors with overexpression in the present study.

In the literature, there are conflicting reports about the correlation between IHC and amplification. For example, Kim *et al.*[18] reported that (4%) of 101 cases scored 1+ had amplification measured by FISH, but Bang *et al.* [ 4] reported in the multicenter ToGA trial that the proportion of IHC 0/1+ samples tested positive by FISH in 594 patients was 23%. In our study, we did not find any amplification sign in IHC 0/1+. We think this discrepancy is likely due to sample sizes and ethnic groups included in the reported studies, but other mechanisms may also contribute the discrepancy, such as polysomy 17 chromosome, altered length in the 5′-untranslated region of mRNA of HER-2/neu, some post-transcriptional events, or the HER-2/neu false positivity of the tumors [19,20].

In our study, the rate of HER-2/neu overexpression/amplification was 11.89%, which fell within the range of 9.3% to 22.6% [12,13,15,18,21-23] reported after 2005 using the old scoring system. It was also in the range of 6% to 12% given by Hsu *et al*. using the new scoring system [24]; these authors were the first to use the new scoring system in a large-scale experiment, with a reported rate of 6.1% for HER-2/neu overexpression. The authors attributed their observed low positivity to the new criteria and some other reasons, such as geographic variations, tumor heterogeneity, the employment of different antibodies, and inter-observer variation. However, one important reason must be considered for the variation in the overexpression, which is what Chung *et al.*[25] found in their screening program for the ToGA: HER-2 positivity rates differed by tumor locations and types, for example, rates were higher in the gastroesophageal junction than in stomach cancer and higher in intestinal than in diffuse or mixed cancer. In the patient group studied by Hsu *et al.*, 16.4% of patients had a tumor of the gastroesophageal junction and 79.8% patients had stomach cancer, but 50.2% had the intestinal type and 49.8% had a mixed and diffused type, which we think was the direct reason for his low positivity rate of 6.1%. The situation was the opposite for Hofman *et al.* [17], who had 71.5% of patients with intestinal type and 28.5% with mixed and diffused type, with a combined HER-2/neu positivity rate of 13.6%. We had a relatively higher proportion of patients with gastroesophageal junction and intestinal type cancer in our patient groups; we thus had a relatively higher proportion of HER-2 positivity rates.

In our study, we found that HER-2/neu overexpression/amplification was associated with Lauren type, WHO histological grade, tumor size and lymph node metastasis. For the association of the HER-2/neu overexpression/amplification with clinicopathological findings, most previous studies reported a higher HER-2/neu overexpression/amplification in intestinal than in diffuse cancers. For example, both Tanner *et al*. [12] and Barros-Silva *et al*. [13] reported similar findings. The ToGA [4] study further confirmed the results by screening a much larger sample size. To date, it is not clear why HER-2/neu is so closely associated with intestinal cancer, but a great number of molecular differences by cancer histology have been reported. For example, E-cadherin was reported to be dominantly confined to the diffuse cancer [26,27]. Intestinal cancer is usually well differentiated, but diffuse cancer is poorly differentiated, which means that overexpression/amplification of HER-2/neu are consistent with WHO classification and Lauren classification. Yonemura *et al.*[28] reported that overexpression of HER-2/neu was related to tumor size, invasion depth, lymph node metastasis and TNM stage, whereas Mizutani *et al.*[29] found that overexpression of HER-2/neu was associated with invasion depth and liver metastasis but not with lymph node metastasis. We believe that the most important reason for differences may be the use of different standards to enroll patients, in addition to selection of tissue samples and the criteria for IHC scoring.

To minimize the impact of staging factor on prognosis analysis, we used patients with curatively resected gastric cancer. In our univariate analysis, overexpression or amplification of HER-2/neu was a prognostic factor but the statistical result for such an association was not present in multivariate Cox proportional hazards model with adjustment of age and sex. In a summary of reported studies on HER-2/neu prognosis after 2005, as shown in Table 4, Park *et al.*[15] also used patients with curatively resected gastric cancer and adopted the old standard and reached the same conclusion as ours after similar analyses, in which they found HER-2/neu to be a prognostic factor in the univariate analysis but not in the Cox proportional hazards model. Later, Hsu *et al.*[24] employed the new standard but failed to yield any positive results. Similarly, Begnami *et al.*[22] also found HER-2/neu amplification to be an independent prognostic factor in univariate log-rank analysis but not in a Cox proportional hazards model.

**Table 4:** Literature review about the prognosis of HER-2 overexpression or amplification in gastric cancer

**Reference**	**N (total/survival analysis)**	**% HER-2/neu+ definition**	**Prognostic factor**	
			**Univariate analysis/association**	**Multivariate analysis**
[12]	131/131	12.2 (FISH+)	Yes/two groups	Not done
[15]	182/182	15.9 (IHC 2+ or 3+)	Yes/two groups	No
Kim MA (2007) [ 18]	248/96	7.7 (FISH+)	Yes/Intestinal type, I stage	Not done
[13]	463/256	9.3 (FISH+)	Yes/Expansive type	Not done
[21]	1414/582 (section), 598/255 (TMA)	12.3 (section), 17 (TMA) (IHC 2+ or 3+)	Yes/Differentiation (W+M)	No (section)/Yes (TMA)
[22]	221/221	15 (FISH+)	Yes/two groups	Not done
[23]	217/217	11 (IHC 3+ )	Yes/two groups and intestinal type	Not done
[24]	1036/1036	6.1 (IHC3+ or IHC2+ and FISH+)	No	No

FISH: fluorescence *in situ* hybridization; IHC: immunohistochemistry; M: moderately differentiated; TMA: tissue microarray; W: well differentiated.

By contrast, Liu *et al.*[23] showed that the overexpression of HER-2 predicted poor prognosis in patients with gastric cancers, but the authors defined IHC 3+ as overexpression and neglected IHC 2+ in their statistical analysis. Worth a mention was that Barros-Silva *et al.*[13], MA Kim *et al.*[18] and KC Kim *et al.*[21] all reached a positive conclusion in their subgroup analysis, especially for the study by MC Kim. They did a subgroup OS analysis in differentiated gastric cancer (well- and moderately differentiated) with the largest sample to date (1,414 patients) and found in the Cox model that Her-2/neu overexpression was a prognostic factor, which perhaps gave us a new perspective to look at Her-2/neu overexpression in gastric cancer. In a time of targeted therapy, how to identify the right target and administrate the right drug to the right patients is a great challenge for both doctors and patients. The ToGA trial led to the conclusion that trastuzumab should be used to treat patients with Her-2/neu overexpression, but according to the above data and analyses, we wonder whether further large studies should be done to refine the right patients for the use of trastuzumab, a drug that is too expensive for every patient to afford in China.

The limitation of this study was the use of microscopy arrays that limited the assessment of HER-2/neu overexpression/amplification to very small portions of the tumor. Although Marx *et al*. [30] reached the conclusion that HER-2 amplification was highly homogenous in gastric cancer, and even a whole-tissue section cannot be guaranteed to contain all the HER-2/neu overexpression/amplification, it is a fact that intratumoral heterogeneity for HER-2/neu overexpression/amplification was found to be more common in gastric cancer than in breast cancer. Thus, our study may underestimate the overall positivity of HER-2/neu overexpression/amplification in gastric cancer.

## Conclusions

We conclud that HER-2/neu overexpression/amplification was present in 11.89% of Chinese patients with gastric cancer, and is not an independent prognostic factor for patients with curatively resected gastric cancer.

## Abbreviations

FISH: Fluorescence in situ hybridization; H & E: hematoxylin and eosin; HER: human epidermal growth factor receptor; IHC: Immunohistochemistry; TMA: tissue microarray; TNM: Tumor-Node-Metastasis; WHO: World Health Organization.

## Competing interests

The authors declare that they have no competing interests.

## Authors’ contributions

FZ carried out the experiment and data acquisition, and drafted the manuscript. NL helped with the experiment and data acquisition. WJ helped with the literature research. ZH and LX participated in collecting the samples. QW helped with writing, discussion of and editing the manuscript. LW designed the study, and read and revised the manuscript. All authors read and approved the final manuscript.
